# *Anopheles gambiae *heat shock protein cognate 70B impedes o'nyong-nyong virus replication

**DOI:** 10.1186/1471-2164-8-231

**Published:** 2007-07-11

**Authors:** Cheolho Sim, Young S Hong, Konstantin A Tsetsarkin, Dana L Vanlandingham, Stephen Higgs, Frank H Collins

**Affiliations:** 1 The Center for Global Health and Infectious Diseases, University of Notre Dame, Notre Dame, IN 46556, USA; 2 Department of Tropical Medicine, Tulane University, 1430 Tulane Avenue SL-17, New Orleans, LA 70112, USA; 3 Pathology Department, Keiller Bldg., 301 University Blvd., University of Texas Medical Branch, Galveston, TX 77555-0609, USA; 4 Department of Entomology, The Ohio State University, Columbus, Ohio 43210, USA

## Abstract

**Background:**

Phylogenetic and functional analysis was conducted on an *Anopheles gambiae *gene, ENSANGG00000017398. Based on phylogenetic analysis, this gene belongs to the same lineage as Heat shock protein cognate 70-4 (Hsc70-4) in *Drosophila*. Accordingly, we propose to name this gene Heat shock protein cognate 70B (HSC70B). We previously reported that expression of HSC70B and other genes including elongation factor-1α (EF-1α) and the agglutinin attachment subunit (agglutinin) were up-regulated in o'nyong-nyong virus (ONNV)-infected female *An. gambiae*. Double-stranded RNA interferences have been applied to further investigate HSC70B, EF-1α and the agglutinin functions in ONNV replication in *An. gambiae*.

**Results:**

Among these three RNAi silenced genes, only dsRNAs of HSC70B (dsHSC70B) promoted ONNV replication in adult *An. gambiae *compared to the control mosquitoes that were co-injected with ONNV and dsRNA of β-galactosidase (dsβ-gal). ONNV titers from mosquitoes co-injected with dsHSC70B were about 9-fold higher at 6 days post-injection (d.p.i.) as compared to the control mosquitoes. By using ONNV tagged with enhanced green fluorescent protein (ONNV-eGFP), co-injection of ONNV-eGFP with dsHSC70B also showed approximately 2 ~ 3-fold higher GFP expression rates than the controls in the head, thorax, and abdomen of the mosquito. Furthermore, co-injection of ONNV with dsHSC70B significantly reduced the lifespan of adult mosquitoes as compared with the control, co-injection of ONNV with dsβ-gal treated mosquitoes.

**Conclusion:**

These results indicate that HSC70B plays important roles in homeostasis and suppression of ONNV replication in the vector, *An. gambiae*. Biological implications of these findings are that while mosquitoes allow ONNV to replicate in them, they also check viral titers so that ONNV infection will result in no harmful effect on mosquitoes. Therefore, mosquitoes can function as vectors of ONNV transmission to humans while ONNV infection in *An. gambiae *remains asymptomatic.

## Background

The arbovirus, o'nyong-nyong virus (ONNV) belongs to genus Alphavirus, and is an enveloped, single stranded, (+) RNA virus with a genome of approximately 12 kb [1,2]. Unlike other arboviruses, ONNV is primarily transmitted by anopheline mosquitoes such as *Anopheles gambiae and An. funestus *[3]. ONNV was first identified during an epidemic in Uganda in 1959, which ultimately infected over 2 million people across East Africa from 1959 to 1961 [4]. Recently it has reemerged in 1996 and 2003 sporadically in Africa [5,6].

Although mosquitoes are critical vectors in many arboviral transmission cycles, there is limited information on how arboviruses influence mosquito gene expression and how mosquito immune systems defend arthropod vectors from deleterious consequences of viral infection. The recent completion of the sequencing of the *An. gambiae *genome has allowed us to investigate modulation of mosquito gene expression resulting from arbovirus infection. Genome-wide screening of differentially expressed transcripts of ONNV-infected female *An. gambiae *relative to naïve females was conducted at 14 day p.i. [7]. Seven genes were identified for their differential expression in ONNV-infected *An. gambiae *compared with controls by cDNA microarrays followed by paired t-test and quantitative real time PCR (qRT-PCR) analysis. The products of the seven genes are seemingly involved in protein translation, DNA replication, or intracellular transport pathways [1]. Among the seven candidates, HSC70B, EF-1α and agglutinin were chosen for further functional studies because of their roles in protein folding, protein elongation, cell adhesion, and cytoprotection, which are all important molecular processes for viral replication.

We hypothesize that elevated gene expression of HSC70B for example, may protect the mosquito cells from ONNV-induced molecular damage [8]. Since molecular chaperons including heat shock protein 70 families regulate protein folding and degradation, it is possible that HSC70B may suppress the non-native viral structural or non-structural protein synthesis in mosquito cells. It was also reported that mammalian and mosquito EF-1α binds to the 3' UTRs of West Nile virus (WNV) and a range of RNA viruses [9-12]. Thus, *Anopheles *EF-1α may have a similar role for ONNV replication in *An. gambiae*. Lastly, agglutinin is a membrane attachment subunit that may interact with ONNV on the membranes of endosomes and lysosomes. Because non-structural proteins and RNAs of alphaviruses including ONNV are associated with the membranes of modified endosomes and lysosomes in the replication complex [13], agglutinin may therefore be involve in a membrane attachment of the replication complex of ONNV.

Based on these assumptions, HSC70B, EF-1α and agglutinin genes were subjected to a detailed functional analysis for their potential involvement in ONNV replication. Using RNAi, we post-transcriptionally silenced target transcripts of the three genes, HSC70B, EF-1α and agglutinin by co-injecting dsRNAs of each target transcript with ONNV into female *An. gambiae*. The result showed that silencing the HSC70B transcript caused significant increase of ONNV titers in female mosquitoes whilst the remaining two genes had no noticeable effects. Herein, we discuss potential antiviral activity of HSC70B in *An. gambiae*.

## Results

### Phylogenetic analysis of 70 Kda Heat shock protein family from *An. gambiae *and *D. melanogaster*

**Figure 1:**
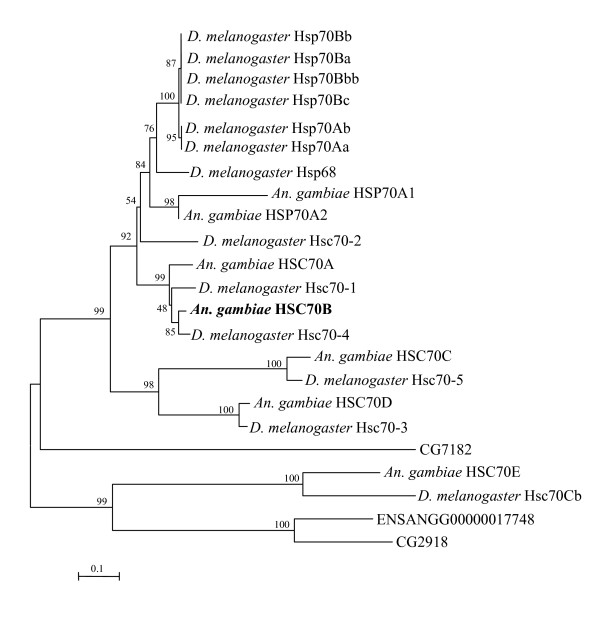
**Phylogenetic tree and multiple sequence alignment of the HSP70 family from *An. gambiae *and *D. melanogaster***. The numbers for interior branches represent bootstrap values. The scale bar indicates an evolutionary distance of 0.1 amino acid substitutions per position. Accession numbers of the nucleotide and amino acid sequences used: *An. gambiae*: HSP70A1, ENSANGG00000001248; HSP70A2, ENSANGG00000022650; HSC70A, ENSANGG00000019768; HSC70B, ENSANGG00000017398; HSC70C, ENSANGG00000016503; HSC70D, ENSANGG00000010404; HSC70E, ENSANGG00000012804; ENSANGG00000017748. *D. melanogaster*: Hsp70Bb, CG31359; Hsp70Bbb, CG5834; Hsp70Bc, CG6489; Hsp70Bb, CG31359; Hsp70Ab, CG18743; Hsp70Aa, CG31366; Hsp68, CG5436; Hsc70-2, CG7756; Hsc70-1, CG8937; Hsc70-4, CG4264; Hsc70-5, CG8542; Hsc70-3, CG4147; CG7182; Hsc70Cb, CG6603; CG2918.

**Figure 2:**
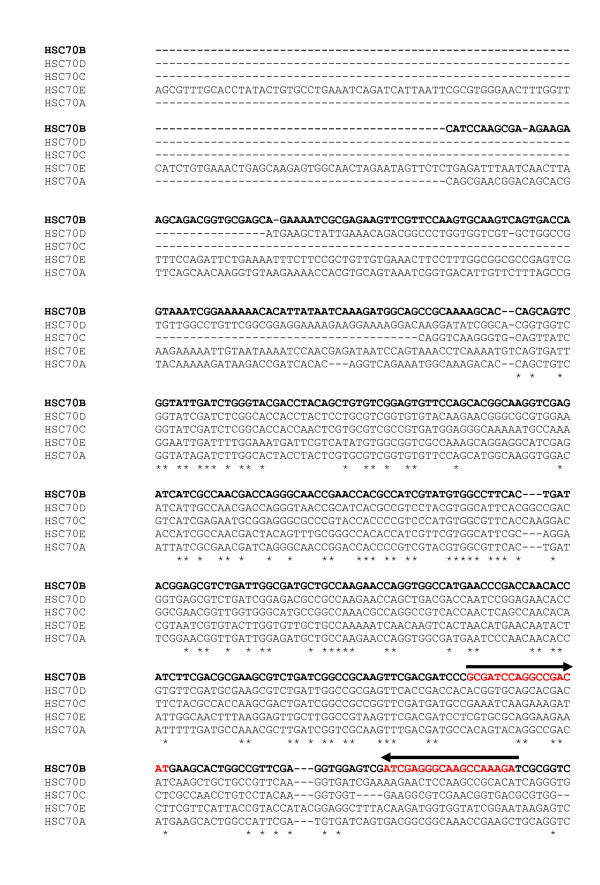
**Multiple sequence alignment of 5' end of coding and non-coding regions of HSC70 family**. The arrows and red characters represent the highly polymorphic sites for the qRT-PCR primers. The bold characters represent the HSC70B gene and the DNA template for the dsRNA of HSC70B. The asterisks denote the conserved sequences among the HSC70 gene family. Accession numbers of the nucleotide and amino acid sequences used: *An. gambiae*: HSC70A, ENSANGG00000019768; HSC70B, ENSANGG00000017398; HSC70C, ENSANGG00000016503; HSC70D, ENSANGG00000010404; HSC70E, ENSANGG00000012804.

**Figure 3:**
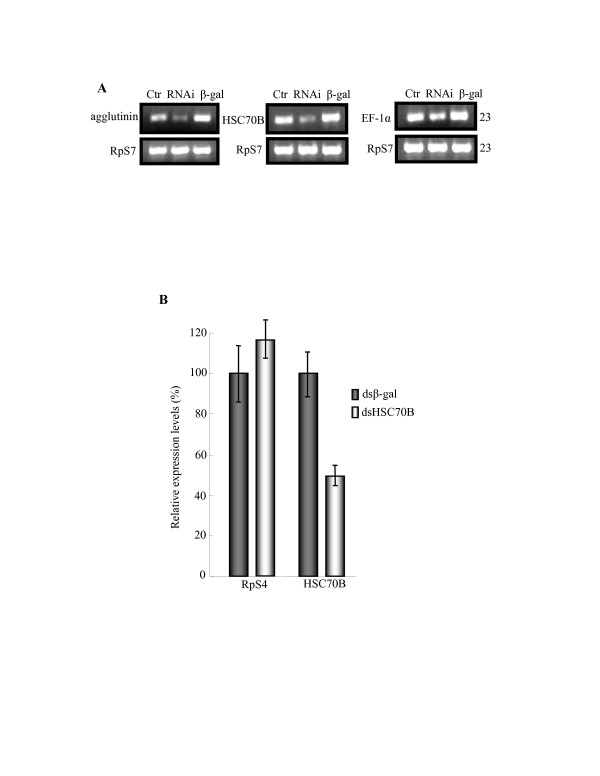
**RNA interference efficiency**. (A) Expression levels were measured by RT-PCR before (Ctr) and 6 days after the respective dsRNA (RNAi) injection based on agglutinin, HSC70B, and EF-1α and *β-galactosidase *(dsβ-gal) as a control. Primers for RT-PCR were designed from agglutinin, HSC70B, and EF-1α as well as ribosomal protein gene *S7 *(RpS7). The expression of RpS7 (23 cycles) served as a loading control. (B) The ribosomal protein gene *S4 *(RpS4) and HSC70B transcript levels (mean ± SD) were measured by quantitative RT-PCR at 6 days after ONNV-eGFP and dsHSC70B and dsβ-gal injections with 3 biological replicates. Primers for qRT-PCR were designed from RpS4 and HSC70B (Table 1). The transcript levels of the loading control (RpS4) did not show significant differences between dsHSC70B and dsβ-gal treatments. However, the HSC70B transcript level in *An. gambiae *with dsHSC70B injection show an average 58% reduction of transcript levels compared to that of the control mosquitoes with dsβ-gal treatment (Student's paired t-test, P = 0.0047).

Phylogenetic analysis of HSP70 genes from *An. gambiae *and *D. melanogaster *indicate that Anopheles HSC70B gene is evolutionarily more conserved with Drosophila Hsc70-1 and Hsc70-4 genes than other Anopheles homologues (Fig 1.). For example, Anopheles HSC70B is most tightly liked to Drosophila Hsc70-4 (Fig. 1). Multiple sequence alignments indicated that there are high polymorphisms near the 5' and 3'-end coding and non-coding regions of the Anopheles HSC70 family (Fig. 2). Indeed, based on these sequence polymorphisms among the Anopheles HSC70 family, we were able to detect unique transcripts of the Anopheles HSC70B gene among the Anopheles HSP70 genes by using the RT-PCR and qRT-PCR primers based on the 3'and 5'-end regions, respectively (Fig. 3 and Table 1). In addition, the dsRNA of Anopheles HSC70B was designed based on the 5' end that was specific to the HSC70B gene, which enabled HSC70B specific knockdown in *Anopheles gambiae*.

**Table 1:** The primer list of qRT-PCR, RT-PCR, and dsRNAs template

Gene ID (GenBank Accession No.)	Primer sequence (5' to 3')	Product size (bp)	Amplification efficiency^c ^(R^d^)
^a^qRpS4 (AJ283756)	Forward: GCTGCCGCTGGTGATCTT Reverse: TCGTCACCTCGCTGTTGGT	65	0.90 (0.99)
qHSC70B (AL930714)	Forward: GCGATCCAGGCCGACAT Reverse: TCTTTGGCTTGCCCTCGAT	64	0.97 (0.99)
EF-1α (XM308429.1)	Forward: CGAGAAGGAAGCTCAGGAGA Reverse: ACGACGACACCTCCTTCTTG	404	
agglutinin (XM311465.2)	Forward: CGGGCGAAACTTACTACAGC Reverse: CGATGGCTACGTTACGGAAT	435	
HSC70B (AL930714)	Forward: GTGAACGAGGCCGAGAAGTA Reverse: TAGTCGACCTCCTCGATGGT	410	
RpS7 (XM_314557.2)	Forward: ATCGCTATGGTGTTCGGTTC Reverse: GCTGCAAACTTCGGCTATTC	627	
E1 (AF079456.1)	Forward: AGAGCCCACACAGCTTCAGT Reverse: ACCGGGTTTGTTGCTATCTG	431	
NS1 (AF079456.1)	Forward: CTTCCTGATGTGCAAGACGA Reverse: ACGACCACAGGCTTGGTATC	440	
^b^dsEF-1α (XM308429.1)	Forward: TAATACGACTCACTATAGGCG AGAAGGAAGCTCAGGAGA Reverse: TAATACGACTCACTATAGGAC GACGACACCTCCTTCTTG	442	
ds-agglutinin (XM311465.2)	Forward: TAATACGACTCACTATAGGCG GGCGAAACTTACTACAGC Reverse: TAATACGACTCACTATAGGCG ATGGCTACGTTACGGAAT	473	
dsHSC70B (AL930714)	Forward: TAATACGACTCACTATAGGTT ACGGTGCGAGCAGAAAAT Reverse: TAATACGACTCACTATAGGCA CGTTCAGTCCCGAGATGT	652	
dsβ-gal (NC000913.2)	Forward: TAATACGACTCACTATAGGGG TCGCCAGCGGCACCGCGCCTTTC Reverse: TAATACGACTCACTATAGGGC CGGTAGCCAGCGCGGATCATCGG	545	

^a^q represent the primer pairs for quantitative RT-PCR, ^b^ds represents the primer pairs of the templates for dsRNA includes T7 promoter for in vitro transcription. ^c^Amplification efficiencies were calculated from the slope of standard curves as *E *= 10^[-1/slope]^-1. 100% PCR efficiency corresponds to an amplification efficiency of 1(Applied Biosystems Application Note); ^d^Regression coefficient of linear standard curve.

### The effect of HSC70B on ONNV replication

First, efficiency of dsRNAi was assessed by using semi-quantitative RT-PCR and qRT-PCR analyses. In contrast to high induction of HSC70B in dsβ-gal and ONNV/ONNV-eGFP coinjected mosquitoes, only traces of HSC70B mRNA were detected in dsHSC70B mosquitoes using semi-quantitative RT-PCR and primers corresponding to the 3' end of the HSC70B gene (Fig. 3A). This result shows that the injection of specific dsHSC70B successfully reduced endogenous target transcripts of HSC70B gene after ONNV injection. Similarly, RT-PCR results also showed the successful post-transcriptional inhibition in agglutinin and EF-1α genes (Fig. 3A).

**Figure 4:**
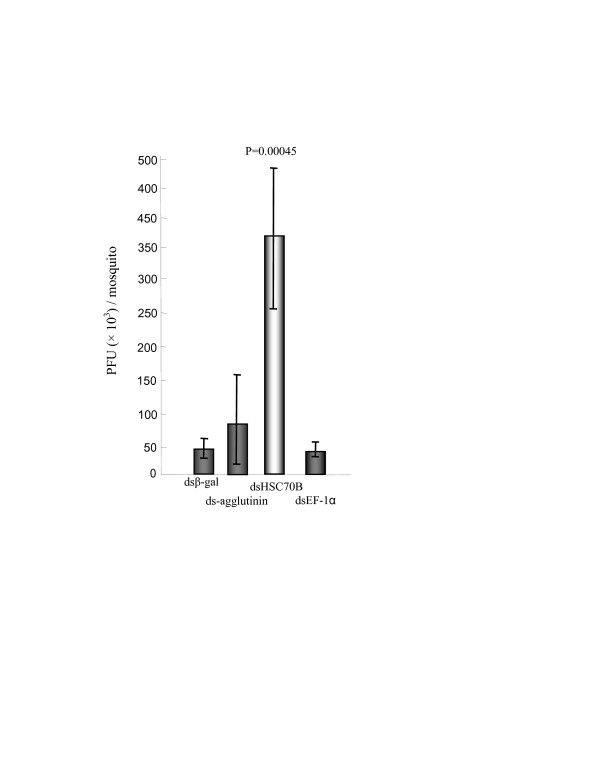
**ONNV titers (mean ± SD) in mosquitoes coinjected with ONNV and the respective dsRNA, including dsβ-gal, ds-agglutinin, dsHSC70B, and dsEF-1α**. ONNV titers in mosquitoes coinjected with ONNV and dsHSC70B had a statistically significant increase compared to that of coinjection of ONNV and dsβ-gal at 6 days p.i. (unpaired t-test, *P *= 0.00045). However, ONNV titers of the dsRNAs of the other genes were not significantly different at 6 days p.i. Each data point was generated by five independent mosquitoes which were co-injected by dsRNA and ONNV.

Mosquitoes were coinjected with ~3.1 × 10^2 ^pfu of virus and 625 ng of the respective dsRNAs targeting the HSC70B, agglutinin and EF-1α gene or the β-gal gene as an internal control (Table 1). To quantify ONNV in the respective dsRNAi-treated mosquitoes, each *An. gambiae *was triturated and ONNV titer was quantified by plaque assay from the treatment groups. Mosquitoes coinjected with ONNV and dsHSC70B had significantly more plaques than those mosquitoes coinjected with ONNV and dsβ-gal (P = 0.00045) at 6 d.p.i. (Fig. 4). No significant differences in the number of plaques were observed among the mosquitoes injected with ONNV and each of ds-agglutinin, dsEF-1α, and dsβ-gal (P ≥ 0.74) (Fig. 4).

**Figure 5:**
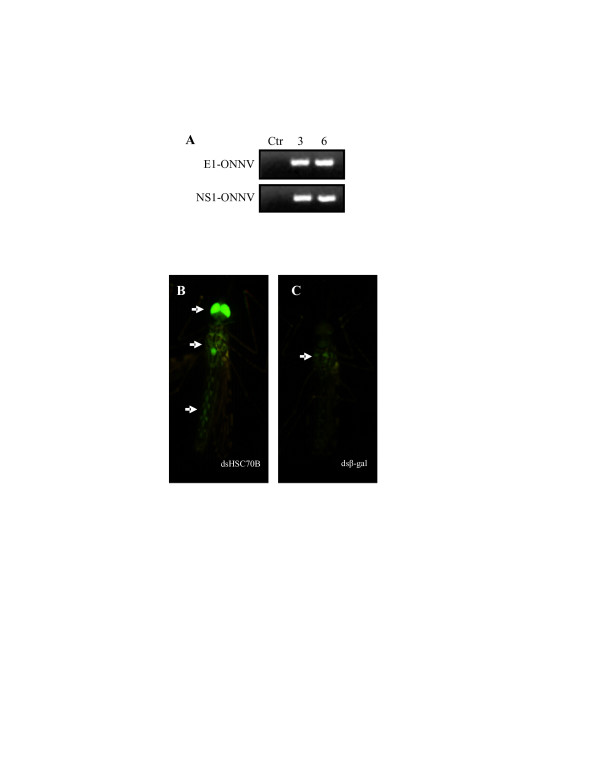
**The phenotype of *An. gambiae *in response to coinjection of ONNV-eGFP and dsHSC70B**. (*A*) Characterization of viral transcripts of ONNV-eGFP after coinjection with dsβ-gal into adult female *An. gambiae *(4arr strain). Ctr, 3 and 6 represent transcript profile of the recombinant ONNV with enhanced green fluorescent protein (ONNV-eGFP) before, at 3 and 6 days p.i., respectively. Primers for RT-PCR were designed from the ONNV structural E1 gene (E1) and non-structural protein 1 gene (nsP1) (Table 1). The PCR products of the E1 and nsP1 primer pairs show the expected sizes, 431 and 440 base pairs, respectively (Table 1), indicating the correct expression of ONNV-eGFP in *An. gambiae*. (B) The strong expression of GFP in head, thorax, and abdomen of *An. gambiae *at 6 days after coinjected with dsHSC70B and ONNV-eGFP. (C) The relatively weak expression of GFP in the thorax of *An. gambiae *at 6 days after coinjected with dsβ-gal and ONNV-eGFP. The arrows indicate the tissues of *An. gambiae *with GFP expression.

To investigate whether up-regulation of HSC70B gene impedes ONNV replication, *An. gambiae *was infected with eGFP-tagged ONNV whilst HSC70B transcripts were silenced by dsRNAi. ONNV titers were then indirectly estimated by visually comparing eGFP expression among three mosquito groups that were coinoculated with dsHSC70B, or dsβ-gal or buffer. In control mosquitoes injected with dsβ-gal or buffer, expression of eGFP was usually weak in head, thorax and abdomen tissues (Fig. 5 and Table 2). Mosquitoes coinjected with ONNV-eGFP and dsHSC70B typically had stronger expression of eGFP in all three tissues (Fig. 5). At 6 dpi, 47% (*n *= 32) of mosquitoes receiving dsβ-gal expressed eGFP in thoracic tissues. However, 87% (*n *= 23) expressed eGFP in thoracic tissues when dsHSC70B was silenced (Table 2). In a similar way, the mosquitoes receiving dsβ-gal showed 38% (*n *= 32) and 22% (*n *= 32) of eGFP expression in head and abdomen, respectively (Table 2). In contrast, mosquitoes receiving dsHSC70B showed 70% (*n *= 23) and 65% (n = 23) of eGFP expression in head and abdomen, respectively (Table 2).

**Table 2:** Percentage of mosquitoes displaying eGFP expression in body tissues after coinjection of ONNV-eGFP and dsHSC70B or dsβ-gal at 6 dpi.

Treatment	Head	Thorax	Abdomen
No dsRNA	54% (20/37)	57% (21/37)	24% (9/37)
dsβ-gal	38% (12/32)	47% (15/32)	22% (7/32)
dsHSC70B	70% (16/23)	87% (20/23)	65% (15/23)

The ribosomal proteins S4 (RpS4) and S7 (RpS7) were used as internal controls for infection studies of ONNV and the malaria parasite, *Plasmodium berghei*, respectively [7,14]. When RpS7 was analyzed by qRT-PCR and cDNA microarray studies, Student's t test determined that the mRNA expression levels of RpS7 in uninfected and ONNV-infected mosquitoes collected at 24 h, 48 h and 14 days p.i. were not significantly different (data not shown). As shown in Figure 3, RT-PCR analysis of RpS7 and qRT-PCR analysis of RpS4 transcript levels at 6 days p.i., detected no significant difference between the relative mRNA levels derived from treated mosquitoes. The results indicate that the low expression levels observed for candidate genes were related to the observed knockdown effect by dsRNAi, and not attributed to variation in sample loading.

### Suppression of HSC70B expression by RNAi and higher ONNV infection level decreases the survival rate of *An. gambiae*

**Figure 6:**
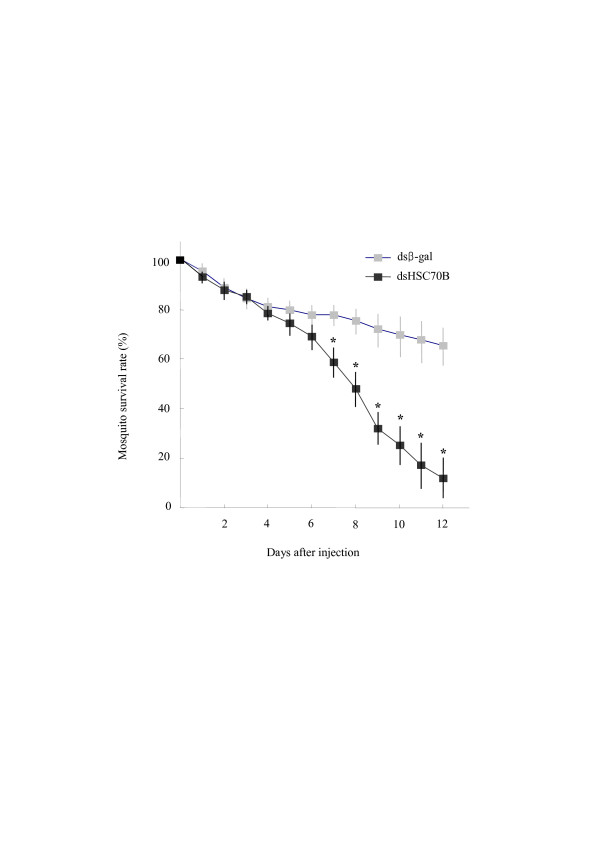
**The relative percentages (mean ± SD) of surviving mosquitoes after the coinjection of ONNV and the respective dsRNA targeting HSC70B and *β-gal *(black and gray, respectively)**. The number of mosquitoes injected represented 100%. Asterisks indicate significant differences in survival (unpaired t-test, P ≤ 0.019). N = 6 groups of 15 individuals for each data point.

Co-injection of ONNV and the dsHSC70B significantly shortened the lifespan of adult mosquitoes compared with the co-injected mosquitoes of ONNV and dsβ-gal, causing a significant reduction of survival rates from 7 days p.i. (Fig. 6, P ≤ 0.019). In addition, *An. gambiae *mosquitoes with down-regulated HSC70B alone lead a reduced survival rate (~80%) at 6 days post injection, though it is much less harmful than co-injection of ONNV and dsHSC70B. This suggests that both reduced expression levels of HSC70B gene and increased ONNV infection level synergistically shorten the lifespan of *An. gambiae *(Fig. 4 and Fig. 6).

## Discussion

In general, the HSP70 family including HSC70B is one of the best studied among the heat shock protein families. The structures of HSP70s and HSC70s are highly conserved in all organisms, from prokaryotes to eukaryotes [15,16]. The conserved region of the molecule consists of an N-terminal adenosine triphosphatase domain (ATPase; ~400 aa), a substrate-binding domain (SBD; ~180 aa), and a carboxyl-terminal domain of variable length and polymorphism [17].

Furthermore, HSC70B, a constitutively induced form of the HSP70 family, was inferred to be located in the cytoplasm [16], and so therefore has may interact with structural and non-structural gene products of ONNV. In fact, from the *in vitro *study with another alphavirus Sindbis, HSC70 purified from bovine brain inhibits the autoprotease activity of Sindbis capsid protein present in the cytoplasm [18].

Interestingly, the prominent antiviral drug cyclopentenone prostanoids and its derivatives, specifically induced HSP70s and HSC70s in treated Vero and mosquito, *Aedes albopictus*, cells [19,20]. Induction of HSP70s has a cytoprotective effect, interrupting viral replication against a wide range of DNA and RNA viruses including Sindbis [20,21]. This antiviral response seems to depend on synthesis of molecular chaperons, which are involved in controlling virus replication in various ways [21]. In addition, recent studies suggested that HSP70 and HSC70 chaperones play a key role in protein quality control, being involved in either folding or degrading of non-native proteins by the ubiquitin-proteasome pathway [22-24].

Alternatively, HSC70B may impede viral replication by modulating other *An. gambiae *proteins that are critical for viral replication in mosquito cells. Since one of the key roles of HSC70B is to regulate nascent unfolded protein as a chaperone [17], the overexpression of HSC70B may shift metabolism of mosquito cells to suppress viral replication. Thus, HSC70B may indirectly repress nascent protein folding of host factors required for ONNV replication in the mosquito. In contrast, a cDNA microarray study in *Ae. aegypti*, which is the principal vector of yellow fever and dengue viruses, reported that Sindbis infection significantly down-regulates the mosquito chaperones, HSP62 and HSP80 [25]. Perhaps, viral suppression of host chaperones may be important for propagation of the virus. Previous studies of the gene products of alphaviruses have shown that these gene products suppress and maneuver host gene expression to favor the viral gene expression system [26,27]. With respect to the regulation/repression of host cell gene expression, there may thus be competition between viral and host factors.

Furthermore, the mosquito and virus may compete for the EF-1α and agglutinin, and this could explain why these proteins are induced upon infection. However, it cannot be ruled out that these knockdown effects may not be sufficient to mimic loss-of-function phenotypes to impair or enhance ONNV replication in *An. gambiae*, because the RNAi technique cannot completely abolish target transcripts. This lack of complete knockout is expected to allow residual expression of target genes, which could result in protein expression that cannot be phenotypically distinguished from wild type or control groups. Therefore proteomics assays measuring viral protein expression in conjunction with EF-1α or agglutinin knockdown will provide more definitive pictures for these proteins' role in ONNV replication in *An. gambiae*.

Suppression of HSC70B by dsRNAi substantially reduced the survival rate of ONNV-infected *An. gambiae *(Fig. 6). In *D. melanogaster*, it has been reported that apoptosis affects primarily cells with the lowest level of HSC70s during embryogenesis, suggesting a role for HSC70s in the control of apoptosis [28]. Several studies have shown that HSP70s and HSC70s binding to the anti-apoptotic protein BAG-1 [29,30]. It has also been reported that HSC70s interacts with the ecdysone receptor and ultraspiracle (EcR/USP) [31], and that high induction of the chaperones extends longevity in *D. melanogaster *and in *C. elegans *[32]. Our data indicates that *An. gambiae *HSC70B is also important for the survival of mosquitoes during viral infection (Fig. 6).

## Conclusion

In summary, our results demonstrate that HSC70B impede ONNV replication in *An. gambiae*. The balance between ONNV propagation and HSC70B abundance may determine, at least in part, the level of ONNV replication and cytotoxicity. Understanding the molecular details of HSC70B interaction with structural and non-structural gene products of ONNV will lead to the elucidation of new approaches to controlling this pathogenic virus. Furthermore, this may suggest new control strategies relying upon the development of transgenic mosquitoes that selectively turn on chaperone genes during arbovirus infection. While molecular mechanisms regarding the regulation of expression of HSP70 genes are relatively well characterized [33], the regulation of HSC70 genes remains to be determined [17].

Our study, in conjunction with the previous microarray experiments, have proven to be effective to identify candidate genes involved in *An. gambiae*-ONNV interactions at the genome level [7]. In addition to genome-wide gene expression studies, an analysis of tissue-specific gene expression is also important, e.g., midgut, fat body, and salivary glands. The discovery of genes that can provide barriers of ONNV transmission in those mosquito tissues will be beneficial to control the viral infection in humans.

## Methods

### Mosquitoes

The 4arr strain of *An. gambiae *was reared at 27°C and 80% relative humidity under a 16 h light: 8 h dark photoperiod, as previously described [34]. Adults were supplied with a cotton wool pad soaked in a 10% sucrose solution *ad libitum *and fed on anaesthetized guinea pigs once per week for egg production.

### Virus

The SG650, strain of ONNV was obtained from the World Reference Center for Arboviruses at the University of Texas Medical Branch, Galveston, TX. Strain SG650 was isolated from human serum in Uganda in 1996 [35] and has been passed once in Vero cells (GenBank Accession Number AF079456). Stock virus was produced following a single passage in Vero cells maintained at 37°C in Leibovitz L-15 medium with 10% fetal bovine serum (FBS), 100 U/mL penicillin, and 100 μg/mL streptomycin. Cell supernatant was harvested when 75% of the cells showed cytopathic effect (3+ CPE). Supernatant containing the virus was collected and titrated. The virus stock contained 2 × 10^7 ^plaque-forming units (pfu)/ml, diluted to 2 × 10^6 ^pfu/ml, aliquoted, and stored at -80°C.

### Generation of ONNV-eGFP

The eGFP gene was amplified from pEGFP plasmid (Clontech Laboratories, Inc) using primers Onn-GFP-Asc-F (5'-GACCTATGGTGAGCAAGGGCGAGGAGCTGTTC-3') Onn-GFP-Pac-R (5'-GACCTTTAATTAATTACTTGTACAGCTCGTCCAT-3'). The PCR product was cloned into AscI and PacI sites of infectious clone pONNic-Foy, provided by K. E. Olson and B. D. Foy (Colorado State University, Fort Collins, CO), which was previously modified by replacing the T7 promoter with a SP6 promoter. pONNic-Foy clone was derived from pONN.AP3, developed by Brault and others [36]. Infectious virus from the infectious clone was produced by linearization with *Not *I, which was *in vitro *transcribed from the SP6 promoter using the mMESSAGE mMACHINE kit (Ambion, Austin, TX) following the manufacturer's instructions. The RNA was electroporated into BHK-21 cells as previously described [37]. Cell culture supernatant containing virus was harvested, aliquoted, and stored at -80°C when cells showed 3 + CPE.

### dsRNA preparation and coinjection into adult female mosquitoes

Templates for the preparation of dsRNA for each candidate gene were PCR-derived fragments flanked by two T7 promoter sequences (TAA TAC GAC TCA CTA TAG) (Table 1). Each PCR-derived fragment were sequenced and blasted against the genomic database of *An. gambiae *[38] to validate the redundancy of the sequence and all confirmed as a unique sequence. Single-stranded RNAs were then synthesized by using the MEGAscript T7 transcription kit (Ambion, Austin, TX) according to the manufacturer's instructions. Annealed dsRNAs were ethanol precipitated and dissolved in injection buffer (0.1 mM sodium phosphate, pH 6.8; 5 mM KCl). In knockdown experiments, ~0.5 μl of a 2:1 mix of dsRNAs (2 μg/μl), ONNV or ONNV-eGFP (~2 × 10^6 ^pfu/ml) were coinjected into the thorax of CO_2_-anesthetized adult females by using a IM 300 Microinjector (Narishige, Japan). Thus, each mosquito was coinjected with ~3.1 × 10^2 ^pfu of virus and ~625 ng of dsRNA.

### Plaque Assays

Each mosquito was triturated in 1 ml of DMEM, and large particulates were pelleted by centrifugation at 300 r.p.m. and then titrated by standard plaque assay in Vero cells [39]. The plaques were counted and the differences in viral titers were analyzed by Pairwise t-tests.

### RT-PCR analysis

Total RNA samples were extracted from three batches of 15 adult female mosquitoes at 6 d.p.i. with ONNV or ONNV-eGFP and the respective dsRNA, using the Trizol Reagent (Invitrogen, CA). To remove genomic DNA contamination, RNA samples were treated with 1.0 μl DNase I following the manufacturer's instructions (50–375 units/μl; Invitrogen, CA). For reverse transcription, 5 μg of total RNA were reverse transcribed with Superscript III RNase H-reverse transcriptase (Invitrogen). Single-stranded cDNAs of different dilution were amplified by PCR using recombinant Taq DNA polymerase (Invitrogen). To show the RNAi efficiency, primers were made to amplify endogenous agglutinin, EF-1α and HSC70b genes of *An. gambiae*. RpS7 gene of *An. gambiae *was used as an internal control for 23 cycles (Table 1). To characterize the ONNV-eGFP transcript, primers were made to amplify endogenous E1 and NS1 genes of for 23 cycles (Table 1).

### Quantitative real-time PCR Analysis (qRT-PCR)

qRT-PCR was performed using an ABI 7700 Sequence Detection System (Applied Biosystems, CA). Standard curves were generated for each transcript tested using 10-fold serial dilutions of *An. gambiae *genomic DNA ranging from 116 to 0.0116 ng per reaction. All reactions were performed in triplicate in a total volume of 25 μl containing 12.5 μl of SYBR Green PCR Master Mix, 300 nmol of each primer at the following conditions: 50°C for 2 min, 95°C for 10 min followed by 50 cycles of denaturation at 95°C for 15 s, annealing and extension at 60°C for 1 min. RNA samples were extracted from mosquitoes at 6 days p.i. Sequences of gene-specific primer sets are given in Table 1. Statistical significance of differences in the expression of individual genes was determined by using a Student's t-test between the relative transcript values derived from the dsHSC70B-injected and dsβ-gal injected mosquitoes across 3 replicates for each gene.

### Survival assay of *An. gambiae *in co-injection of dsHSC70B and ONNV

To evaluate the knockdown effect of HSC70B gene on the survival rate of ONNV infected *An. gambiae*, 15 females per cohort were intrathoracically co-injected with dsHSC70B and ONNV. For control, 15 females per cohort were intrathoracically coinjected with dsβ-gal and ONNV. Each treated cohort was kept in 8 cm (diameter) ×12 cm cages with a cotton wool pad soaked a 10% sucrose solution. The cages were placed at 27°C and 80% relative humidity under a 12 h light: 12 h dark photoperiod, and mosquito survival was assessed at 24 hours. Survival was defined as the ability of the mosquito to right itself. Experiments for each of the two groups were replicated six times.

### Sources of Sequence Data

The *An. gambiae *genome has 10 genes containing the HSP70 domain [40]. Among these, two genes which have a short fragment of HSP70 domain were excluded for further analysis (ENSANGG00000023531, ENSANGG00000023619). The HSP70 gene sequences for *Drosophila *were obtained from the Berkeley Drosophila Genome Project [41,42].

### Multiple Sequence Alignments and Phylogenetic Tree Construction

Multiple sequence alignments were performed by using ClustalW v1.81 [43]. Phylogenetic trees were constructed by the Neighbor-Joining (NJ) and maximum parsimony (MP) methods, both included in MEGA3 [44]. The accuracy of reconstructed trees was examined by the bootstrap test with 10,000 replications.

## Authors' contributions

CS carried out the study with contributions from YSH, TK and DLV. CS drafted the manuscript with contributions from YSH, KT, DLV, SH and FHC. All authors read and approved the final manuscript.
